# Stigma trajectories, disclosure, access to care, and peer-based supports among African, Caribbean, and Black im/migrant women living with HIV in Canada: findings from a cohort of women living with HIV in Metro Vancouver, Canada

**DOI:** 10.1186/s12889-024-20439-3

**Published:** 2024-11-13

**Authors:** Faaria Samnani, Kathleen Deering, Desire King, Patience Magagula, Melissa Braschel, Kate Shannon, Andrea Krüsi

**Affiliations:** 1 Department of Medicine, University of British Columbia, 317 – 2194 Health Sciences Mall, Vancouver, BC V6T 1Z3 Canada; 2 Centre for Gender and Sexual Health Equity, University of British Columbia, 1190 Hornby St, Vancouver, B.C V6Z 2K5 Canada; 3 Afro-Canadian Positive Network of BC, 10318 Whalley Blvd, Surrey, BC V3T 4H4 Canada; 4 School of Criminology, Simon Fraser Univeristy, 8888 University Dr W, Burnaby, BC V5A 1S6 Canada

**Keywords:** Stigma, HIV disclosure, African, caribbean, and black women, Peer-based support, Access to care

## Abstract

**Background:**

African, Caribbean, and Black im/migrant women experience a disproportionate burden of HIV relative to people born in Canada, yet there is scarce empirical evidence about the social and structural barriers that influence access to HIV care. The objectives of this study is to estimate associations between African, Caribbean, and Black background and stigma and non-consensual HIV disclosure outcomes, and to understand how experiences of stigma and im/migration trajectories shape access to HIV care and peer supports among African, Caribbean, and Black im/migrant women living with HIV in Canada.

**Methods:**

This mixed-methods analysis draws on interviewer-administered questionnaires and semi-structured interviews with self-identifying African, Caribbean, and Black women living with HIV in the community-based SHAWNA (Sexual Health and HIV/AIDS: Women’s Longitudinal Needs Assessment) cohort. Bivariate and multivariable logistic regression using generalized estimating equations (GEE) were performed to estimate associations between African, Caribbean, and Black background and stigma and non-consensual HIV disclosure outcomes. Drawing on a social and structural determinants of health framework, qualitative analysis of interviews elucidated the interplay between migration trajectories, stigma, racialization, and HIV.

**Results:**

Amongst our participants (*n* = 291), multivariable GEE analysis revealed that African, Caribbean, and Black participants (*n* = 15) had significantly higher odds of recently being outed without consent as living with HIV (AOR 2.34, 95% CI 0.98–5.57). Additionally, African, Caribbean, and Black participants had higher odds of recent verbal or physical abuse due to their HIV status (AOR 2.11, 95% CI 0.65–6.91). Reflecting on their im/migration trajectories, participants’ narratives (*n* = 9) highlighted experiences of political violence and conflict, trauma, stigma, and discrimination associated with HIV in their place of origin and the racialization and stigmatization of HIV in Canada. Fear of disclosure without consent was linked to barriers of accessing care and peer-based supports.

**Conclusion:**

Our findings indicate that im/migration trajectories of African, Caribbean, and Black women living with HIV are critically related to accessing HIV care and supports in Canada and compound HIV stigma and discrimination. HIV disclosure without consent complicates access to care and social/peer support, underscoring the need for privacy, confidentiality, and the importance of building trust in the context of clinical encounters. The results of this study emphasize the critical need for culturally sensitive trauma-informed care models rooted in peer-based approaches.

## Highlights

• African, Caribbean, and Black im/migrant women living with HIV in Canada experience a disproportionate risk of HIV disclosure without consent
• Experiences of political violence, trauma, racism, stigma, and discrimination are linked to barriers to accessing HIV care
• Increased efforts to ensure privacy and confidentiality are important towards building trust in the context of clinical encounters
• Collaboration with the African, Caribbean, and Black community is imperative to developing culturally sensitive, trauma-informed HIV services rooted in peer-based approaches

## Background

Over the past decade the incidence and prevalence of HIV among women have decreased slightly in Canada [1]. In 2020 about one in four people living with HIV were women and an estimated 32% of new HIV diagnoses were among women [1]. Racialized women, including African, Caribbean and Black and Indigenous women (including First Nations, Métis, and Inuit) are disproportionately affected by HIV in Canada. According to most recent data from the Public Health Agency of Canada from 2014, African, Caribbean, and Black women represent 35% of reported HIV cases in 2014 [2] yet Black people constitute only 4.3% of the Canadian population [3].

### *African*,* Caribbean*,* and Black women*,* Immigration*,* and HIV*

Although data shows that African, Caribbean, and Black women are overrepresented among women living with HIV in Canada, substantially less is known about how culturally appropriate and accessible HIV services can be tailored with and for im/migrant African, Caribbean, and Black women in their place of residence. African, Caribbean, and Black women face unique individual, social, and structural challenges throughout their resettlement journey into Canada [4, 5]. UNAIDS cites HIV-related stigma and discrimination as one of the chief barriers to accessing services along the HIV cascade of care globally [6, 7]. While HIV-related stigma is highly prevalent across the globe, it is culturally specific, varies between different settings and is mediated by cultural and moral beliefs, structural barriers, stereotypes, religion, and gender inequities [8–11]. Previous research has highlighted how HIV stigma in some African settings is shaped by limited access to health care, as well as, the communality and close-knit nature of communities [8]. Social isolation and loneliness are commonly experienced upon im/migration therefore communities can provide a great source of strength and support in which immigrants can lean on. Consequently, HIV disclosure without consent can present great barriers where depending on the community in which one resides, people living with HIV may experience feelings of shame, embarrassment, and fear of ostracization if their HIV status is exposed [8].

As part of the Canadian immigration process, all potential immigrants and refugees are forced to undergo mandatory HIV testing and therefore encounter additional challenges that affect physical and mental well-being [12]. These challenges include cultural, socio-political, and economic barriers, such as stress related to managing a potential new diagnosis, risk of gender-based violence, fear of not being able to immigrate to Canada due to HIV status, language barriers, mistrust of health care providers, social isolation and food insecurity [4, 5]. Furthermore, those receiving a positive test result are at higher risk of experiencing emotional or physical abuse upon disclosure to their partners [13, 14]. The process of disclosure is especially complicated by Canada’s assertive approach to criminalizing HIV, whereby people living with HIV must use a condom AND have a low or undetectable viral load, or risk criminal liability for not disclosing status to their sexual partners [15]. African, Caribbean, and Black women living with HIV also need to navigate HIV-related stigma and discrimination in Canada; racist stereotypes entrenched in the contextualization of HIV as an “African” disease continue to permeate HIV discourse [7, 16]. As a result, African, Caribbean, and Black women living with HIV continue to experience high levels of HIV-related stigma [16]. Ultimately, the intersection between stigma, gender, and racial discrimination alongside Canada’s reinforcement of HIV exceptionalism by criminalizing HIV non-disclosure poses significant barriers to disclosing HIV status in order to receive care, treatment, and support [15, 17–19].

Existing literature and research have focused primarily on understanding the current experiences of African, Caribbean, and Black women living with HIV in Canada. However, it is critically important to understand women’s im/migration trajectory from pre- to post-migration with a focus on social and structural barriers to care that shape access to HIV treatment and supports. As Oxman-Martinez et al. [4, p. 394] state, “women’s health is perceived as a continuum that extends throughout the lives of women, critically and intimately related to their life condition”. The stigma and discrimination of having been diagnosed and living with HIV in another country in addition to the trauma inherent in the immigration process forms the backdrop for interacting with the health care system in Canada [20, 21]. It is critically important to understand this intersection of identities in order to implement health care practices that respond to the increasing HIV incidence of African, Caribbean, and Black women in Canada [22]. Therefore, the focus of this study is to estimate associations between African, Caribbean, and Black background and stigma and non-consensual HIV disclosure outcomes, and to understand how experiences of stigma and im/migration trajectories shape access to HIV care and peer supports among African, Caribbean, and Black im/migrant women living with HIV in Canada.

## Methods

### Study design and population

This mixed-methods study is informed by a social determinants of health conceptual framework which is used to conceptualize the underlying socioeconomic, political, and structural processes including housing, work environment, social support, stress, nutrition, and physical activity that underpin health inequities [23, 24]. In our study, this framework was used to understand the social, structural, political, and economic factors that affect access to HIV health care and services amongst African, Caribbean, and Black im/migrant women living with HIV. This study draws on longitudinal cohort data and qualitative interview data collected in the context of the Sexual Health and HIV/AIDS: Women’s Longitudinal Needs Assessment (SHAWNA) project. SHAWNA is an open longitudinal, participatory, and community-based research project and includes a quantitative and qualitative arm both focused on examining the social, political, legal, gender, and geographic barriers to care in cis and trans women’s sexual health and HIV care across Metro Vancouver, Canada. The project was initiated after extensive community consultations with women living with HIV, HIV care providers, community-based organizations, and policy experts in order to examine the social, structural, and systemic gaps in cis and trans women’s sexual health and HIV care. Guided by a Community Advisory Board comprised of local and national community organizations and a Positive Women’s Advisory Board of 12–15 women living with HIV from Metro Vancouver, the project is committed to “MIWA/MIPA” principles of greater and meaningful involvement of people living with HIV. Inclusion criteria consisted of being 14 years old or over, and identification as either a cis or trans woman living with HIV and currently residing or accessing HIV support services in Metro Vancouver.

### Quantitative data Collection

Data collected in January 2010 to February 2019 were drawn from SHAWNA for the current study sample. Data from January 2010 to August 2014 were drawn from An Evaluation of Sex Workers Health Access (AESHA), a cohort of sex workers in Metro Vancouver, which has a harmonized survey with the SHAWNA Project. 27% of participants in SHAWNA were also enrolled in AESHA.

Women in SHAWNA were recruited through community outreach staff, self-referrals, and referrals from HIV care providers, peer navigators, HIV/AIDS organizations, and clinical outreach. Following informed consent, women completed an interviewer-administered questionnaire by trained community interviewers at enrolment (between January 2010 and February 2019) and semi-annually (every 6 months). The questionnaire consisted of a wide range of topics including individual (ex. age, gender/sexual identity, ethnicity), biological (ex. CD4 cell counts), social (ex. trauma, violence, social support, sex work), and structural variables (ex. homelessness, financial support). At each visit, participants also completed voluntary viral load/CD4 testing and serology for sexually transmitted infections/Hepatitis C Virus serology by one of the project’s sexual health nurses. Treatment was offered by project nurses onsite, if needed, for symptomatic STI infections and Papanicolaou testing, regardless of enrolment in the study. Participants received an honorarium of $50CAD at baseline and each biannual visit for their time, expertise, and travel. This study holds ethical approval from the Providence Health Care/University of British Columbia Research Ethics Board and BC Women’s Hospital. Data was securely collected and managed using REDCap electronic data capture tools hosted at UBC [25, 26].

### Quantitative variables

#### Primary variable of interest

The primary variable of interest in our quantitative analysis was a time fixed measure for identifying as African, Caribbean, and Black (reporting a racial background as African, Caribbean, or Black vs. all other racial backgrounds, including White, Indigenous (First Nations, Métis, Inuit), and otherwise racialized persons). This was used as the main explanatory variable in our analysis. Participants were able to respond to multiple options representing racial/ethnic identities in the survey. While we recognize great heterogeneity in race/ethnicity across participants, and also how intersecting identities may shape health and health services access in complex ways, it was necessary for us to make decisions about how to categorize participants with multiple identities for the purposes of quantitative analysis. Participants were included as African, Caribbean or Black if they selected a racial/ethnic identity of African, Jamaican or Black. Two participants identified as both Indigenous (First Nations, Métis or Inuit) and African, Caribbean or Black; these participants were included as Indigenous. If participants selected any other racial/ethnic identity in addition to White, they were included as the former racial/ethnic identity. These decisions were made due to practical considerations regarding smaller sample sizes among some racial/ethnic identities and restrictions of the analytic approach, and were guided by anti-oppression principles acknowledging colonial violence experienced by all Indigenous Peoples alongside shared experiences related to unjust and inequitable social, structural and systemic racism, discrimination experienced by Black, Indigenous and other racialized people in Canada.

#### Outcomes

Two outcomes measuring different aspects of stigma were examined: having HIV status disclosed without consent (i.e. “In the last six months, has anyone “outed” you for knowing or suspecting you were HIV positive?”, yes or no), and verbal/physical abuse due to HIV status (i.e. “In the last six months, has anyone verbally or physically abused you for knowing or suspecting you were HIV positive?”, yes or no). Both outcomes were time updated at each semi-annual study visit. In addition, lifetime experiences of these outcomes were examined descriptively.

#### Other variables of interest and confounders

Time-fixed socio-demographic factors examined in our analysis included: being born in Canada (vs. im/migrating to Canada); English fluency (defined as most comfortable speaking English vs. most comfortable speaking another language); education (defined as high-school graduate vs. less than high school); sexual orientation (sexual minority at any study visit (LGBTQ vs. heterosexual) and gender identity (cisgender woman = gender identity matches biological sex at birth vs. trans woman = anyone who identity as a woman differs from their biological sex at birth including trans women, trans sexual or other transfeminine identities). Participants were given the option of providing more than one response to questions on sexual orientation, gender identity, and racial identity. Based on evidence that minority stress processes affect gender and sexual minority communities to a greater extent than cisgender and heterosexual people [27], we examined the impact of identifying as a sexual and/or gender minority woman, compared to those who only identify as cisgender and heterosexual women.

All other variables were time-updated to capture events in the last six months or current measures at each semi-annual study visit. These included interpersonal and structural variables based on their known or hypothesized relationship with the explanatory variable of African, Caribbean, and Black identity. Interpersonal variables included age (in years), ever experiencing physical/sexual violence (yes or no), and ever exchanging sex for money/goods/services (yes or no). Structural variables included ever been homeless or living on the street (yes or no).

We considered potential confounders in the relationship between African, Caribbean, and Black racial identity and the two outcomes based on previous research and a priori knowledge. These included recently parenting a child (yes or no), current cohabitation with other(s) (i.e. answering > 0 to “How many other people are living in the same place with you?”), access to or use of HIV-specific health and support services (yes or no), unstable housing (i.e. homelessness, single-room occupancy hotels, staying with parents, family, or other relatives etc.; vs. supportive housing or own apartment/house), living in HIV specific supportive housing (yes or no), having had an intimate male partner (yes or no), and reporting that HIV is having negative physical effects (i.e. fatigue, nausea, night sweats, weight loss, stomach/abdominal pain, other).

### Qualitative data Collection

In the qualitative phase, experienced community and peer interviewers living with HIV conducted in-depth interviews between 2015 and 2017 with 64 SHAWNA cohort participants who were invited to participate in semi-structured interviews during their bi-annual cohort study visit. Sampling of participants for the qualitative arm aimed to represent the demographic variation of women living with HIV in Metro Vancouver. Given the focus of the current analysis on the experiences of African, Caribbean, and Black im/migrant women, we included all African, Caribbean, and Black participants who participated in the qualitative arm of the project (*n* = 9). All African, Caribbean, and Black cohort participants who could be reached to be inivited to participate in semi-structured interviews chose to participate during the qualitative data collection phase 2015–2017 (*n* = 9).

The qualitative interview guide was developed collectively with women living with HIV and HIV service providers and focused on understanding social and structural factors that shaped participants’ lives. These issues included access to healthcare and social services, sexual health, and HIV-related stigma. The primary focus of the interview guide included exploring overall health and wellness, experiences with HIV treatment, sexual and reproductive health, parenting, HIV stigma, housing, and incarceration. Prior to conducting the semi-structured interviews, all participants provided informed written consent. Each interview lasted approximately 60 to 120 min and participants were remunerated with a CAD $30 honorarium for their time, expertise, and travel. The study holds ethics approval by the Providence Healthcare/University of British Columbia Research Ethics Board – H14-01073.

### Data analyses

#### Quantitative

Descriptive statistics at baseline were calculated for sample characteristics, including for continuous (means, medians, interquartile ranges) and categorical (prevalence, percent) variables. These interpersonal, behavioural, and structural variables were stratified by identifying as African, Caribbean, and Black, and differences were examined using Pearson’s chi squared tests (or Fisher’s exact test for small cell counts) for categorical variables and the Wilcoxon rank-sum test for continuous variables. Bivariate and multivariable odds ratios (ORs and AORs) with 95% Confidence Intervals (CIs) and two-sided p-values were calculated using logistic regression with generalized estimating equations (GEE) and an exchangeable working correlation matrix to estimate associations between identifying as African, Caribbean, and Black and the two outcomes of interest: HIV status disclosure without consent and verbal/physical abuse due to HIV status. An exchangeable correlation matrix structure was chosen based on the assumption that responses from the same individual are equally corelated, regardless of the ‘distance’ (in this case, amount of time between follow-up surveys) between responses. Logistic regression using GEE were used to account for repeated measures among participants over time, and potential confounders were included in the multivariable models. SAS version 9.4 was used for all statistical analyses (SAS Institute Inc., Cary, North Carolina, USA).

### Qualitative

All audio recordings of in-depth interviews were transcribed verbatim and checked for accuracy by long-time, highly experienced contractors. Due to the focus of this study on African, Caribbean, and Black im/migrant women, interview transcripts by participants who self-identified as being of African, Caribbean, and Black descent (*n* = 9) were included in the Qualitative analysis. Data were analyzed thematically and included both inductive and deductive methods [28, 29]. Data analysis was guided by a social determinants of health framework [28]. Transcripts were initially coded using a deductive approach based on four main themes from the interview guide that focused on migration and mobility. These codes included reasons for migrating, experiences with the Canadian health care system, barriers to care, living positively in Canada as a woman living with HIV. In an iterative process, the lead author (FS) applied a second round of inductive codes that emerged from the data and drew on a social determinants of health framework, including structural and systemic factors to elucidate how women living with HIV negotiate HIV disclosure, stigma, and discrimination [23]. These codes included experiences of political violence, experiences of intimate partner violence, spirituality as a coping mechanism, and experiences of social isolation. Coding was conducted using Nvivo qualitative data analysis software. Potentially identifying information from participants’ quotes was removed. Triangulation was used to establish credibility and congruence in our findings; specifically, our coding framework, analysis, and results were reviewed by staff members with shared identities, including living with HIV and an im/migration background, additionally the qualitative findings are complimented by also drawing on quantitative results [30].

The qualitative interviews with participants who self-identified as African, Caribbean, and Black women living with HIV was analyzed in combination with the respective quantitative data. This mixed-methods approach transcends the traditional quantitative and qualitative research methods in order to offer a more “informative, complete, balanced, and useful research results” [31, p. 129]. As Johnson et al. explain, there are various frameworks and definitions of mixed-methods research; however, our approach increases the depth and breadth of our findings by integrating both quantitative and qualitative data on experiences with HIV stigma and non-consensual HIV disclosure into a single study [31].

## Results

### Quantitative results

Our study sample of the overall cohort study included 291 participants with 1618 observations over 8.5 years from August 2010-February 2019. There were 52 observations over this period among African, Caribbean and Black participants. Socio-demographic characteristics of the study sample participants are described in Table 1.

**Table 1:** Baseline demographics and characteristics of women living with HIV stratified by African, Caribbean or Black identity

	Total N = 291 (100)	Identify as African, Caribbean, and Black N = 15 (5.2)	Do not identify as African, Caribbean, and Black N = 276 (94.8)	P-value
Age (median, IQR)	44 (37–51)	43 (36–54)	44 (37–50)	0.698
Sexual and/or Gender minority identity^a^	108 (37.1)	5 (33.3)	103 (37.3)	0.756
Most comfortable speaking English	276 (94.8)	9 (60.0)	267 (96.7)	< 0.001
High school graduate	137 (47.1)	9 (60.0)	128 (46.4)	0.303
Ever been homeless/living on street	233 (80.1)	7 (46.7)	226 (81.9)	0.003
Ever exchanged sex for money/goods/services	222 (76.3)	7 (46.7)	215 (77.9)	0.010
Any physical/sexual violence by any perpetrator, ever	277 (95.2)	14 (93.3)	263 (95.3)	0.421
Ever had HIV status disclosed without consent	143 (49.1)	10 (66.7)	133 (48.2)	0.194
Ever verbally/physically abused due to HIV status	104 (35.7)	8 (53.3)	96 (34.8)	0.160

Data derived from SHAWNA cohort (2010–2019)

All data refer to n (%) of participants unless otherwise specified

IQR: interquartile range

^a^Inclusive of sexual minority identity (inclusive gay, lesbian, bisexual, Two spirit, asexual, queer, other) and/or gender minority identity (inclusive of trans, intersex, transexual, two spirit, genderqueer, other) vs. cisgender and heterosexual.

At baseline, the median age for African, Caribbean, and Black participants (*n* = 15) was 43 (IQR: 36–54). Amongst these 15 participants, 33% of African, Caribbean, and Black participants identified as a sexual minority (lesbian/ bisexual / queer women vs. heterosexual women), 60% of African, Caribbean, and Black participants were comfortable speaking English, and 60% of African, Caribbean, and Black participants had graduated from high school. Over the course of the study, 47% of African, Caribbean, and Black participants had their HIV status outed without consent, and 40% were verbally or physically abused due to their HIV status.

In bivariate logistic regression using GEE (Table 2), women with an African, Caribbean, and Black background had higher odds of being outed as HIV positive without consent in the last 6 months (Odds Ratio (OR): 2.26, 95% Confidence Interval (CI): 0.96–5.28), and being verbally or physically abused due to their HIV status within the last 6 months (OR: 2.56, 95% CI:0.99–6.60). In multivariable logistic regression using GEE (Table 2), after adjusting for hypothesized confounders, having an African, Caribbean, and Black background had higher odds of being outed as living with HIV without consent within the last 6 months (AOR 2.34, 95% CI 0.98–5.57). In multivariable analysis, African, Caribbean, and Black participants also experienced higher odds of being verbally/physically abused due to HIV status in the last 6 months (AOR 2.11, 95% CI 0.65–6.91).

**Table 2:** Odds ratios (OR) from bivariate and multivariable GEE for outcomes associated with African, Caribbean, and black identity

Outcome	Unadjusted OR (95% CI)	Unadjusted *p*-value	Adjusted OR (95% CI)	Adjusted *p*-value
HIV status disclosed without consent^a^	2.26 (0.96–5.28)	0.061	2.34^b^ (0.98–5.57)	0.056
Verbally/physically abused due to HIV status^a^	2.56 (0.99–6.60)	0.052	2.11^c^ (0.65–6.91)	0.216

Data derived from SHAWNA cohort (2010–2019)

^a^Time updated at each visit to capture events in the last six months

^b^Adjusted for hypothesized confounders, including recent (in the last six months) unstable housing, recent HIV specific supportive housing, currently cohabiting with other(s), recently accessing any HIV health or support services in Metro Vancouver, recently parenting a child, and reporting that HIV is having negative physical effects.

^c^Adjusted for hypothesized confounders, including recent (in the last six months) HIV status disclosure without consent, recent unstable housing, recent HIV specific supportive housing, recently having an intimate male partner, and reporting that HIV is having negative physical effects.

### Qualitative results

These quantitative findings were supplemented and contextualized with qualitative interviews from the nine women living with HIV who self-identified as African, Caribbean, and Black drawn from the larger set of 67 SHAWNA qualitative interviews conducted between 2015 and 2017, in order to further understand the stigma and discrimination experienced amongst this community. The median age of African, Caribbean, and Black participants in the qualitative interviews was 47 years with a range from 24 to 68. Five participants were parents with a range of one to six children; furthermore, all participants identified as cisgender, resided in stable housing, and accessed HIV care within Metro Vancouver.

Participants interview narratives emphasized three key components, each centered on the relationship between HIV disclosure and stigma, that were pivotal in African, Caribbean, and Black im/migrant women’s lives. Each shaped their experience living with HIV, accessing care, and receiving support in Canada. We categorized these components as (1) migration to Canada, (2) navigating the Canadian health care system, and (3) social support and resilience.

### Migration to Canada

Participants’ narratives revealed homogenous motives for im/migrating to Canada. The effects of war, conflict, and political violence forced participants to im/migrate leaving behind unsafe living conditions for themselves and their families. As described by P1 and P2:

*“I came to Canada just because of the political situation and my husband was arrested. I was also kidnapped […] It was around election time I was kidnapped*,* because they wanted to- they were raping girls all the time.” (P1)*.

*“And the war came […] And there where I was. [.] We suffer a lot. Struggling and asking United Nation to help us; to resettle us. And our day throughout- the struggle was too hard.” (P2)*.

Many participants also spoke about sexual violence they had encountered either directly from their partner, or from other men in the community. For some, this was how they contracted HIV. As described by P2 and P9:

*“I was raped*,* […]*,*. They have war conflict*,* too*,* in their country. And that’s where I got the sickness.” (P2)*.

*“I don’t understand how. Why did they rape me? There were so many people*,* why did they come to my place? We were so nice to everybody.” (P9)*.

The close connection between having experienced sexual violence and contracting HIV heightened the shame many participants felt and further perpetuated the stigma and discrimination they experienced. Participants’ narratives emphasized the very serious and negative repercussions of HIV stigma, including further abuse and violence, that compounded and intensified the fear and anxiety of HIV disclosure without consent. For many, it was impossible to live within their place of origin without continually fearing for their lives if their HIV status were disclosed. P5 and P6 described the dire consequences of HIV stigma and discrimination in detail:

*“Oh if I stayed in* [country of origin] *I think I could not be there now. I could be dead because*,* as I told you*,* uh in in* [country of origin], *people frustrate people so much when they hear that you are HIV.” (P5)*.

*“But this is the problem. Like especially if I tell them that I am HIV*,* me- They’ll they gonna kill me before I die. I will go to the grave*,* before I go. Before God call me that […] it’s your time. My life would be hell.” (P6)*.

As evidenced by the multivariable findings in which African, Caribbean, and Black women had higher odds of being outed as living with HIV, the collective impact of these traumatic events surrounding HIV stigma and the very severe consequences of HIV disclosure, were linked to heightened fear of being disclosed as living with HIV in Canada. The ramifications of discrimination, negative societal attitudes, and alienation not only formed the backdrop of participants’ navigation of the Canadian health care system, but also shaped participants’ access to services and supports and ultimately their overall health and wellbeing.

### Navigating the Canadian Health Care System

HIV remains highly stigmatized not only in the participants places of origin, but also in Canada. Emotions such as shame and guilt were commonly experienced upon receiving an HIV diagnosis; therefore, accessing HIV care that was supportive and comprehensive was vital for participants upon entry into Canada. As participants reflected on commencing their journey in Canada, it became clear that for many this was their first time directly experiencing racialization, racism and “othering” based on their skin colour. These experiences, in addition to the pervasive social construction of HIV as a Black or African disease by mainstream media and institutions, further marginalized African, Caribbean, and Black women living with HIV. The consistent “othering” of African, Caribbean, and Black women living with HIV and experiences of racism compounded the anxiety and fear participants felt about what others thought or felt about them and consequently shaped their initial perception of Canada. As explained by P1 and P8:

*“That people*,* you know*,* when you are inside for the busses…you know*,* the white people they just thinking the African people*,* all of them they have got HIV. Because when you sit in the seat*,* they don’t like sit with you. But when the bus is full of- they come and sit with you.” (P1)*.

*“I know that it’s different because it’s a white man country. I know that I would be different. I know the change*,* it would be there. My skin*,* already*,* has changed. Like Africans*,* as you know*,* are black. I was struggling sometime with the HIV.” (P8)*.

The process of HIV disclosure is complicated. Disclosure is often framed as being beneficial as it allows patients to access treatment and support while also counteracting stigma and discrimination present within the community [32, 33]. This was illuminated in our research as several positive factors associated with HIV disclosure, primarily centered around accessing HIV-specific and women-centred care, were discussed. P3 and P4 shared their experiences accessing a woman and family-centred HIV clinic:

*“Yeah. It’s like my family to me because*,* specifically when you get*,* you have HIV positive you are you’re not accepted mostly you know.” (P3)*.

*“One good thing about* [the HIV clinic] *is that you see everyone [different care providers] in one day. You don’t have to go today*,* tomorrow.” (P4)*.

Although there are a variety of health care services available across Metro Vancouver, the process of accessing HIV care presents with several risks and barriers especially considering the traumatic experiences that participants encountered in their places of origin. The interlinking nature of HIV stigma persistent in both African communities as well as in Canada constituted a major barrier to care for many women. Multiple and intersecting forms of discrimination, rooted in the experiences prior to migration and racist stereotypes experienced in Canada, exacerbated both the perceived and enacted stigma that participants experienced. As initially conveyed in our quantitative analysis, the accessibility of HIV-specific services was limited by fear of inadvertent disclosure of HIV status to members of the community, by merely being seen in the vicinity of an HIV-specific service or clinic. As P3 described:

*“Some women they’re in hiding. They don’t want to be seen when they go to the clinic […]Because not all black people like to be… Once they meet you* [at an HIV-specific service] *you know the whole community is going to know.” (P3)*.

P7 further explained how the perception of HIV within her country of origin prevented her from accessing a counsellor in Canada out of fear that her status would be disclosed, and that she would be ostracized:

*“It’s just*,* where I come from*,* when people find out you’re positive*,* they kind of treat you like a reject and you’re dead*,* “You want to kill us*,* you want to kill us”*,* and you’re like*,* alone. You don’t talk to people about it because you know how they’re going to react*,* so the best way is just to keep it inside.” (P7)*.

To keep their HIV status secret, many women were hesitant to access HIV services out of fear of encountering someone they recognized. This was especially the case in situations where HIV services were located in open waiting areas in hospitals. P2 explained her fear and concern regarding her HIV diagnosis being spread back to the community:

*“You know*,* we are black. She will tell the other people that came way from other countries*,* they don’t know. And when she tell them [about my HIV status] start going back to the country that I come from*,* it will not look good.” (P2)*.

In a context of pervasive HIV-related stigma, previous negative disclosure experiences in addition to fears that their HIV status may get shared back to the country they moved to Canada from acted as a significant barrier to accessing HIV-services. Intense anxiety and concern over inadvertent HIV disclosure threaten participants likelihood of accessing health care services or adhering to treatment and medication and can result in detrimental outcomes for both themselves and their family’s lives.

### Social support and resilience

In contrast to the harmful consequences of disclosure that participants encountered in the past and continually feared, many consistently stressed the importance of social and peer-led supports in helping to share their stories and create a supportive environment for women living with HIV. Based on prior negative experiences, many women were hesitant to disclose their HIV status let alone discuss it within a group setting. Given the fact that timing of disclosure is highly dependent on social contexts, the first interaction was vital in helping create a safe space where the women felt more comfortable sharing their own experiences living with HIV, exchanging information, and addressing concerns. P4 described the relief she experienced following the first interaction with her peer support group:

*“The first day I was sharing my story it was a retreat and then that’s when I just felt comfortable sharing my story and also it was a safe place for all the women. So that was a good change.” (P4)*.

Furthermore, participants spoke to the sense of belonging and community they felt from having a peer support group. P3 and P4 described this in detail:

*“If you’re HIV you*,* you have no nothing. People have nothing to do with you*,* we are not accepted in the community. That’s why we keep ourself like supporting each other as women and*,* which is good […] This uh*,* organization [African*,* Caribbean*,* and Black HIV support group] it has really helped us a lot. Bringing us together and making us feel at home or we meet and cry and cope and*,* hug each other and*,* you know we share*,* ideas and we move on like that. Because the this is like our family here. We don’t have anybody else.” (P3)*.

*“[We need a space where] we can share our stories*,* we cry together in a safe place*,* and then we support one another. Like we pay visit for one another*,* so I always babysit with those kids. We help one another like family*,* like extended family. So we call one another ‘brother’*,* ‘sister’” (P4)*.

The significance and need for social support groups were further emphasized given recent cuts in government and program funding for HIV support organizations. Social supports are vital for people living with HIV; therefore, the recent defunding of over 40 programs and services by the Public Health Agency of Canada (PHAC) and the federal Canadian government are highly concerning. P3 discussed the impact these cuts had on her and the community:

*“Because we don’t have funding but once in a while we used to come out like in the sunny day and we’d go to the park and entertain the kids*,* cook*,* eat together*,* which was very meaningful. But now we don’t have anything like that.” (P3)*.

In the context of pervasive stigma, women living with HIV may not be comfortable presenting their HIV status in certain circumstances due to prior negative experiences both in their country of origin and upon entering Canada. However, disclosure and peer support as reflected in the above narratives, also presents an opportunity to increase access to social support systems and create a shared supportive environment that can help women reclaim power and resilience.

Alongside peer support, the majority of participants stressed the importance of utilizing coping mechanisms such as spiritual practices, to overcome stigma and discrimination. As P6 explained when asked to give advice to a newly diagnosed woman in a similar situation:

*“Um the advice I will give her*,* is to be strong. Not to think about this HIV anyway. Because there is a lot of disease*,* who can kill people. Not HIV alone. HIV if you take good care of you*,* of yourself*,* it will not kill you […] You have to be happy.” (P6)*.

P8 and P9 further described the benefits and importance using spiritual practices as a source of resilience and strength:

*“God made me like this*,* to be here*,* in Canada today. […] God let me live another day*,* “Yes*,* she’s sick”. Sometimes I think that God is with me*,* no*,* he kick me – made me strong.” (P8)*.

*“There were times when I was upset*,* when I asked God why I should live. But that has passed. I used to not want to wake up*,* just once. But I am still alive*,* still here…He said to stay who you are*,* where you are. I am with you. I will watch over you.” (P9)*.

The initial perception and stigmatization these women had about HIV were contradicted by their narratives surrounding their current relationship with this disease. Many had come to terms with their diagnosis and continue to use it as a source of strength to survive and thrive amidst adversity. Participants coping strategies were rooted in longstanding significance placed on community and in particular one local grass roots community organization that supports people living with HIV and religious practices.

## Discussion

In summary, our findings demonstrate that in the context of pervasive HIV-related stigma, fear of HIV disclosure both pre- and post-migration presents a significant barrier to accessing care and treatment for African, Caribbean, and Black im/migrant women living with HIV. The HIV disclosure process is highly intricate and affected by many factors including but not limited to ethnicity, gender, culture, age, and class. As recounted by many participants, stigma and discrimination associated with being outed as living with HIV without consent spanned from experiences in their place of origin and continued upon im/migration to Canada. Many women reported the complex conditions under which they lived in their place of origin due to the risk of ostracization, abuse, or violence if their status was disclosed.

Through mixed-methods analysis, our findings highlight that African, Caribbean, and Black women disproportionately experienced HIV disclosure without their consent and were more likely to experience HIV-related verbal or physical violence. Our qualitative findings outline how fear of HIV disclosure and stigma can contribute to barriers to accessing HIV care and peer support programs. Our findings build upon previous work by understanding the im/migration journeys of African, Caribbean, and Black women from their place of origin and upon im/migration to Canada. Although the social and structural factors that contribute to African, Caribbean, and Black women contracting HIV in their home countries are well-established, there is limited research into understanding how these experiences translate into the ongoing HIV stigma that continues once relocated into Canada, and how this negatively impacts access to social support and HIV treatment [15, 16]. Our findings highlight the importance of understanding the history of traumatic stress, fear, and distress that compound the stigma and discrimination African, Caribbean, and Black women experience, and the potential it has to interfere with accessing medical care, adhering to HIV treatment, or engaging in social support.

As highlighted from our quantitative results, there was evidence that African, Caribbean and Black women living with HIV had higher odds of being outed as living with HIV without consent and experiencing verbal and physical abuse relative to women who did not identify as African, Caribbean and Black. The close connection between being outed as living with HIV without consent and experiencing verbal and physical abuse emphasize the unintended consequences associated with HIV disclosure. This was further elucidated by our qualitative results whereby HIV disclosure shaped the relationship that women had with their diagnosis, and in turn their interaction with the Canadian health care system and social support from family and community. Through mixed-methods analysis, our findings build upon previous work by understanding the im/migration journeys of African, Caribbean, and Black women from their place of origin and upon im/migration to Canada. Although the social and structural factors that contribute to African, Caribbean, and Black women contracting HIV in their home countries are well-established, there is limited research into understanding how these experiences translate into the ongoing HIV stigma that continues once relocated into Canada, and how this negatively impacts access to social support and HIV treatment [17, 18].

Our focus on the continuum of women’s lives and migration journeys highlighted the interconnected nature of stigma and discrimination experienced within the place of origin and the continual racialization of HIV within Canada. Multiple intersecting and overlapping forms of HIV-related stigma occurred at the interpersonal, community, economic, and structural levels, both at the place of origin and upon im/migration, which interfered with accessing medical care, adhering to HIV treatment, or engaging in social support [34]. Interpersonal factors of traumatic stress, fear, and distress co-occurred alongside community ostracization and sexual violence, which was further shaped by structural level stigma rooted in cultural norms and systems. The stigma process at multiple levels continued to shape participants ability to engage in HIV care and treatment upon im/migration into Canada. The complexity of HIV-related stigma highlights the importance of implementing public health strategies that consider the intersection of interpersonal, social, and structural factors that shapes health and wellbeing [34]. This is especially important in order to develop health care strategies that optimize HIV care outcomes amongst African, Caribbean, and Black women living with HIV.

Our findings further elucidate the complexities and circumstantial nature of HIV disclosure. As emphasized through participants’ narratives, disclosure without consent can result in harmful and severe consequences that can be highly devastating for women living with HIV including gender-based violence, ostracization, and stigma and discrimination both within healthcare and in the community [16, 21]. In contrast, other instances such as in the presence of peers and social support groups present opportunities for women living with HIV to disclose their status in order to interact and form supportive relationships with others in similar circumstances [35]. Extensive research has shown that peer support provided within a confidential setting is a vital resource for people living with HIV from a variety of cultural backgrounds and settings [21, 36–39]. For example, in Zambia people living with HIV indicated interest in peer support programs despite not having disclosed their HIV status to others, similarly in China research has documented positive experiences with peer counselling despite strong concern for privacy [38, 39]. The vast success of peer support programs for people living with HIV has been recognized by various organizations across Canada who continue to support African, Caribbean, and Black women living with HIV despite drastic funding cuts [40]. Overall, our findings highlight complexities of HIV disclosure; they stress the importance of navigating these difficult scenarios in which women elucidate the importance of maintaining privacy and confidentiality regarding HIV status while also desiring a peer-based social support group in which they can share similar experiences. This is important because it substantiates the use of culturally sensitive and trauma-informed care specific for African, Caribbean, and Black women living with HIV that are modeled through peer-based approaches.

The practice of trauma-informed care to inform HIV prevention and treatment strategies has gained increased attention and emphasis [41]. Trauma-informed care refers to understanding the longitudinal impact of traumatic events as not having simply occurred in the past, but as shaping a person’s current health, treatment, and recovery [42, 43]. Therefore, past traumatic experiences can influence the interaction, response, and relationship individuals have with the health care system and their health care providers [42, 44]. The primary goal of trauma-informed care is to foster a sense of safety, collaboration, transparency, and trust while reassuring patients of their right to privacy as health care providers seek to learn more about the patient’s experiences with trauma in order to elucidate potential barriers to HIV care [41]. This includes but is not limited to routine and universal trauma screening, compassionate and appropriate responses to trauma disclosure, safety planning, and referrals to community resources [44, 45]. The overall benefit of this model is to provide optimal care within a trusted health care setting where patients feel empowered, engaged, and secure in making decisions and taking actions affecting their own health.

Our research has demonstrated that trauma and stress is compounded for women having endured the resettlement process while also living with HIV, and that they have higher odds of HIV status being disclosed without consent and abuse related to their HIV status. Given the disproportionate burden of HIV incidence among African, Caribbean, and Black women and the history of stigma and discrimination experienced, our findings further emphasize the need for trauma-informed and culturally sensitive care tailored towards the needs and experiences of African, Caribbean, and Black women living with HIV. Implementing a longitudinal approach that targets multiple aspects of the resettlement process, from pre- to post-migration, is instrumental towards addressing the severe ramifications of having experienced war related trauma, receiving a positive HIV diagnosis, and the trauma of migrating and adapting to a new setting. Once in Canada, improving HIV programs to focus on the heightened stigma and discrimination experienced by African, Caribbean, and Black women is vital towards promoting disclosure of HIV status in order to access health care [46]. Furthermore, working closely with African, Caribbean, and Black women to understand and respond to cultural differences in terms of HIV care and treatment can pave the way for providing effective transcultural care [22, 46]. Improving access to education focused on culturally appropriate care is urgently needed for health care providers to understand how to appropriately communicate and engage patients in their care. Lastly, our findings highlight that framing this model of care around a peer-based approach will be pivotal. A peer-based trauma-informed model of care can be crucial in helping African, Caribbean, and Black women navigate living with HIV, act as a bridge towards seeking care and treatment, and support the healing process [35–39, 44].

Our study design had several limitations. First, our quantitative sample size of African, Caribbean, and Black participants may have been too small to detect all statistically significant associations, though repeated measures among participants were used to increase statistical power. Furthermore, the generalizability of findings was reduced by our small sample size, and the fact that our cohort is not a representative sample of all women living with HIV in Metro Vancouver or of women living with HIV in other settings. For example, there were no trans African, Caribbean, or Black women living with HIV in the qualitative portion of the study. However, to substantiate our findings, we complimented our quantitative analysis with in-depth interviews to corroborate our results, while also gaining a breadth and depth of understanding. Additionally, we drew on a community-based participatory approach that includes staff members and interviewers with lived experience of im/migration and HIV and have built strong relationship between interviewers and participants, to further increase the credibility of our results.

## Conclusion

Our findings indicate that the pre- and post-migration trajectories of African, Caribbean, and Black women living with HIV compounds HIV stigma and discrimination, further precluding African, Caribbean, and Black women from disclosing their HIV status and accessing health care services. This study highlights the importance of adapting our current health care model by creating culturally sensitive trauma-informed services rooted in peer-based approaches. The recent funding cuts by PHAC and the federal government is a step in the wrong direction as it limits the development of innovative services tailored to the specific needs of the African, Caribbean, and Black community.

## Data Availability

All relevant data are presented within the paper and are fully sufficient to replicate the study findings.

## Abbreviations

HIV: Human Immunodeficiency Virus
AIDS: Acquired immunodeficiency Syndrome
GEE: Generalized estimating equations
SHAWNA: Sexual Health and HIV/AIDS: Women’s Longitudinal Needs Assessment
UNAIDS: Joint United Nations Programme on HIV/AIDS
MIWA/MIPA: Meaningful Involvement of Women Living With HIV/AIDS
PRA: Peer Research Associate
LGBTQ: Lesbian, Gay, Bisexual, Trans, Queer
CI: Confidence Intervals
AOR/OR: (Adjusted) Odds Ratio
IQR: Interquartile Range
PHAC: Public Health Agency of Canada
